# miRNAs and Alzheimer’s Disease: Exploring the Role of Inflammation and Vitamin E in an Old-Age Population

**DOI:** 10.3390/nu15030634

**Published:** 2023-01-26

**Authors:** Virginia Boccardi, Giulia Poli, Roberta Cecchetti, Patrizia Bastiani, Michela Scamosci, Marta Febo, Emanuela Mazzon, Stefano Bruscoli, Stefano Brancorsini, Patrizia Mecocci

**Affiliations:** 1 Institute of Gerontology and Geriatrics, Department of Medicine and Surgery, University of Perugia, 06132 Perugia, Italy; 2 Department of Medicine and Surgery, University of Perugia, 05100 Terni, Italy; 3 Department of Medicine and Surgery, Section of Pharmacology, University of Perugia, 05100 Terni, Italy; 4 IRCCS Centro Neurolesi “Bonino-Pulejo”, Via Provinciale Palermo, Contrada Casazza, 98124 Messina, Italy; 5 Division of Clinical Geriatrics, NVS Department, Karolinska Institutet Stockholm, 17177 Stockholm, Sweden

**Keywords:** aging, inflammation, oxidative stress, miRNAs, vitamin E

## Abstract

Alzheimer’s disease (AD) is the most frequent cause of dementia worldwide and represents one of the leading factors for severe disability in older persons. Although its etiology is not fully known yet, AD may develop due to multiple factors, including inflammation and oxidative stress, conditions where microRNAs (miRNAs) seem to play a pivotal role as a molecular switch. All these aspects may be modulated by nutritional factors. Among them, vitamin E has been widely studied in AD, given the plausibility of its various biological functions in influencing neurodegeneration. From a cohort of old-aged people, we measured eight vitamin E forms (tocopherols and tocotrienols), thirty cytokines/chemokines, and thirteen exosome-extracted miRNAs in plasma of subjects suffering from subjects affected by AD and age-matched healthy controls (HC). The sample population included 80 subjects (40 AD and 40 HC) with a mean age of 77.6 ± 3.8 years, mostly women (45; 56.2%). Of the vitamin E forms, only α-tocopherol differed between groups, with significantly lower levels in AD. Regarding the examined inflammatory molecules, G-CSF, GM-CSF, INF-α2, IL-3, and IL-8 were significantly higher and IL-17 lower in AD than HC. Among all miRNAs examined, AD showed downregulation of miR-9, miR-21, miR29-b, miR-122, and miR-132 compared to controls. MiR-122 positively and significantly correlated with some inflammatory molecules (GM-CSF, INF-α2, IL-1α, IL-8, and MIP-1β) as well as with α-tocopherol even after correction for age and gender. A final binary logistic regression analysis showed that α-tocopherol serum levels were associated with a higher AD probability and partially mediated by miR-122. Our results suggest an interplay between α-tocopherol, inflammatory molecules, and microRNAs in AD, where miR-122 may be a good candidate as modulating factor.

## 1. Introduction

Alzheimer’s disease (AD) and related dementia represent the main causes of severe disability in old age subjects, which strongly impact human and financial costs. According to the age of onset, AD can be classified into two categories: early-onset AD, whose development starts before, and late-onset AD after 65 years [1]. These disorders have a heterogeneous origin, and several markers have been established as risk factors for early onset AD, where genetic aspects play a more impactful role [2]. Despite decades of studies, the underlying etiopathogenetic mechanisms of AD are still unknown, and no cure is available. Recent evidence suggests that the late-onset form, like other chronic conditions, may develop under multiple factors and can be considered a geriatric syndrome [3]. To date, only symptomatic treatments have been promoted and available, including anticholinesterase inhibitors and memantine. Thus, the finding of novel and potential “disease-modifying” drugs able to reduce the progression or interfere with pathogenic mechanisms of such a disease represents an area of intense research.

In the brain pathology of AD, oxidative stress and inflammation play an essential role, representing important features of this disease. Alterations due to oxidative damage can be detected in cell membranes (such as lipid peroxidation), proteins (such as post-translational changes) as well as in nucleic acids [4,5,6]. Inflammatory changes include the activation of brain cells (microglia and astrocytes) with high production and increased levels of pro-inflammatory factors, including cytokines [7]. Interestingly, a state of sub-clinical and persistent inflammation is considered a cause of dysregulation of mechanisms able to clear damaged proteins in the brain [8]. Moreover, high levels of inflammatory molecules have been detected in subjects affected by AD both in the brain and plasma [9]. In this context, nutritional factors strongly influence several pathophysiological processes in AD, with significant implications for nutrition in preventive or therapeutic strategies. So, a healthy lifestyle based on a correct diet seems to be a powerful strategy for preventing or reducing AD progression [10]. Furthermore, many biologically active nutritional factors have been found to modulate oxidative and inflammatory processes in vitro and in human studies [11]. For example, several nutrients with antioxidant properties, which include vitamin E, have been shown to reduce the risk for AD promotion and dementia progression [12]. Vitamin E is the main lipid-soluble, chain-breaking, non-enzymatic antioxidant in the human body. It is represented by a family of eight natural forms that include four tocopherols and four tocotrienols classified into the α, β, γ, and δ forms. Vitamin E is mostly consumed through the diet and present in various foods (including wheat germ oil, sunflower, soybean oil, almonds, peanuts, beet greens, collard greens, spinach, mango, and avocado), while the main active form in tissues is α-tocopherol [12]. 

A recent study showed that nutrients through the habitual diet might influence circulating microRNA (miRNA) profiles [13]. MiRNAs are a class of small non-coding RNAs playing a relevant epigenetic role in controlling gene expression and may represent mediators between dietary intake and health status. A previous study demonstrated that exosomal synaptic proteins in the blood could predict the development of AD five to seven years before its clinical onset [14]. However, this model requires the detection of multiple proteins, which makes this process complex and expensive. A subsequent study showed a method to predict preclinical AD using miRNAs in plasma exosomes, which is relatively simple and less expensive [15]. In addition, some identified miRNAs (i.e., miR-155, miR-146, and miR-223) were shown to play an essential role in regulating acute inflammatory factors in the innate immune response [16,17].

Evaluating the whole circulating miRNome by small RNA-sequencing performed on plasma samples of healthy volunteers with different dietary habits (vegans, vegetarians, and omnivores), a recent study showed that vitamin E correlated with the most significant number of miRNAs [13]. Among vitamin E forms, α-tocopherol has been proposed in AD treatment mainly due to its antioxidant and anti-inflammatory capacities [18].

To the best of our knowledge, only a limited number of studies have explored the relationship between vitamin E serum levels, inflammatory molecules, and circulating exosomal miRNAs. Since, as recently suggested, miRNAs control inflammation and oxidative stress [18], both altered in AD, a link with vitamin E cannot be ruled out. Considering such evidence, we investigated a potential interplay between vitamin E, inflammation, and microRNAs in a group of old-age subjects with or without AD.

## 2. Materials and Methods

### 2.1. Subjects and Study Design

This cross-sectional study was conducted on 80 old age subjects selected based on the following inclusion criteria.

#### 2.1.1. Healthy Control (HC)

Age and education adjusted Mini-Mental State Examination (MMSE) score ≥ 27; no active neurological or psychiatric disorder; no ongoing medical problems or related treatments interfering with cognitive function; a normal neurological exam; no psychoactive medications; the ability to live and function independently in the community.

#### 2.1.2. Alzheimer’s Disease (AD)

AD was diagnosed according to standard research criteria [19] and classified as mild AD (CDR 1, according to the Clinical Dementia Rating). AD diagnosis was confirmed by a combination of clinical and neuropsychological evaluation (assessing different cognitive areas such as memory, language, and constructional praxis) and brain imaging (3T Magnetic Resonance Imaging-MRI). In both groups, individuals with elevated plasma inflammatory markers -as serum C-reactive protein (CRP), erythrocyte sedimentation rate (ESR), and white blood cell (WBC) count- or evidence of acute inflammatory or infectious diseases, diabetes, malignancies, immunologic or hematologic disorders or treatment with anti-inflammatory drugs (including aspirin or NSAID in the last three months, even in a single dose) were excluded from the study. The CRP, ESR, and WBC cut-off were ≥0.5 mg/dL, 35 mm 1° h, and 9.6 × 10^3^, respectively. After a clear explanation of the study, all subjects provided written informed consent to participate in the research approved by the institutional review board of the University Hospital.

### 2.2. Cognitive, Functional, and Neuropsychological Assessment

Cognitive performances were assessed with a neuropsychological battery as previously described [20] that included the MMSE-with a large battery of specific tests evaluating different cognitive areas; the Clinical Dementia Rating scale (CDR) used to rank dementia severity; and the Geriatric Depression Scale (GDS) assessed current depressive symptoms. In addition, an informant-based rating of functional status was carried out using the Activity of Daily Living (ADL) and the Instrumental Activity of Daily Living (IADL) scales. A higher score indicates better-preserved self-sufficiency.

### 2.3. Blood Sample

At the recruitment, blood samples were collected in EDTA tubes from a peripheral vein after overnight fasting and kept immediately on ice. Plasma was separated by centrifugation (4000 rpm for 15 min at 4 °C), aliquoted, and stored at −80 °C until analyzed.

### 2.4. Vitamin E

Plasma tocopherols and tocotrienols levels were measured with reverse-phase high-performance liquid chromatography (HPLC) using an electrochemical CoulArray system (ESA, Chelmsford, MA, USA). Aliquots of 200 μL were mixed and extracted three times with a 1:2 ratio of ethanol to hexane, concentrated to dryness with high-purity nitrogen gas, and reconstituted in 300 μL mobile phase. β-Tocopherol (Superchrome, Milan, Italy); α-, γ-, and δ-tocopherol, α-, γ-, and δ-tocotrienol (LGC-Promochem, Milan, Italy), β-tocotrienol (Matreya-DBA, Pleasant-Gap, PA, USA) were used as standards. After filtration, analyte separation was conducted at room temperature on a Discovery-C18-column (Sigma-Aldrich). The mobile phase (30 mmol lithium acetate/L, 83% HPLC grade acetonitrile, 12% HPLC grade methanol, and 0.2% HPLC grade acetic acid, pH 6.5) was delivered at 1 mL/min [21].

### 2.5. Inflammatory Molecules

A multiplex biometric ELISA-based immunoassay was used according to the manufacturer’s instructions (MILLIPLEX MAP Human Cytokine/Chemokine Magnetic Bead Panel–Immunology Multiplex Assay, Millipore). The following molecules were measured: EGF, EOTAXIN, G-CSF, GM-CSF, INF-α2, IFN-ɤ, IL-10, IL-12p40 IL-12p70, IL-13, IL-15, IL-17, IL-1RA, IL-1α, IL-1β, IL-2, IL-3, IL-4, IL-5, IL-6, IL-7, IL-8, IP-10, MCP-1, MIP-1α, MIP-1β, TNF-α, TNF-β, VEGF, RANTES. Measurements were performed in duplicate. The analytes concentration was calculated using a standard curve, with software provided by the manufacturer (Bio-Plex Manager Software).

### 2.6. miRNAs

Exosome precipitation and isolation were performed with miRCURY™ Exosome Isolation Kit– Serum and plasma. 500 μL of plasma samples were centrifuged a second time (3200× *g*, 5 min, 4 °C) to exclude cellular debris, including whole cells and membrane fragments, which was then transferred into a new tube. A total of 6 μL of Thrombin (stock concentration of 500 U/mL) were added to the plasma fraction; the sample was incubated for 5 min at room temperature and spun for 5 min at 10,000× *g*. A total of 200 μL of Precipitation Buffer was added to the sample and incubated for 60 min at 4 °C. After centrifugation for 5 min at 500× *g*, 270 μL of Resuspension Buffer was gently mixed with the residual pellet. The purified exosome sample was collected after final centrifugation of 5 min at 500× *g*. At this point, 1 μL of Exiqon RNA spike-in mix (UniSp2, UniSp4, and UniSp5) as quality control was added.

Total RNA, including miRNAs, was extracted with miRCURY™ RNA Isolation Kit -Biofluids. After ethanol addition, the solution was loaded to the Mini Spin Columns. Centrifugation of samples allowed RNA to be retained by the filter while all other contaminants were washed away. After three washing steps, RNA was eluted through the addition of Elution Buffer. Total RNA was quantified with Qubit 2.0 (Qubit™ microRNA Assay Kit, Thermo Fisher Scientific, Waltham, MA, USA). Eight nanograms of RNA diluted with nuclease-free water were reverse transcribed with miRCURY LNA™ Universal RT microRNA PCR (Exiqon, Vedbaek, Rudersdal, Denmark). The total reaction volume was 20 μL (4 μL of Reaction Buffer, 2 μL of Enzyme Mix, 1 μL of RNA spike-in control UniSp6 as a positive cDNA synthesis control). Samples were kept at 42 °C for 60 min, and cDNA was immediately used for real-time PCR assays with ExiLENT SYBR^®^ Green Master Mix (Exiqon system). Stratagene Mx3005P instrument (Agilent Technology, Santa Clara, CA, USA) was used for PCR assay. 4 μL of cDNA diluted 1:20 with nuclease-free water were used in a total reaction volume of 10 μL. Melting curve analysis was carried out, each sample was run in triplicate, and results were averaged; no template controls were included in the analysis. RNU6 was used to normalize data. The −ΔCt method was used to calculate the relative expression of the target genes as follows: −ΔCtmiRNA= −(Cttarget − CtRNU6).

### 2.7. Statistical Analysis

The observed data were normally distributed (Shapiro–Wilk W-Test) and are presented as means ± Standard Deviation (SD). To assess differences between groups, unpaired Student’s *t*-test or Pearson’s Chi-squared (χ^2^) test were used, as appropriate. Simple and partial Pearson’s correlation analyses were also performed, as indicated. Binary logistic regression analyses were performed to assess the association between α-tocopherol levels and dementia, controlling for multiple covariates. All *p* values are 2-tailed, and the level of significance was set at *p* ≤ 0.05. Statistical analyses were performed using the SPSS 26 software package (SPSS, Inc., Chicago, IL, USA) and GraphPad Prism 6.0 (GraphPad Software, San Diego, CA, USA).

## 3. Results

### 3.1. Sample Characteristics

The sample population included 80 subjects (40 HC and 40 AD) with a mean age of 77.6 ± 3.8 years (age range: 70-85 years old), primarily women (45; 56.2%). Table 1 shows the descriptive and biochemical characteristics of the studied population. The subjects affected by AD were mainly women (27F/13M; χ^2^ = 4.114, *p* = 0.035), while in the HC group were men (18F/22M) with a statistically not significant difference. HC group was younger than AD (76 ± 4 vs. 79 ± 4 years, *p* = 0.002). As expected, subjects affected by AD had lower MMSE, ADL, and IADL scores. No other differences were found.

### 3.2. Vitamin E

Among the Vitamin E forms, only α-tocopherol (22.24 ± 2.25 vs. 24.63 ± 2.76 µmol/L, *p* < 0.0001) differed between groups having AD significantly lower levels as compared with HC independent of gender (22.32 ± 0.40 vs. 24.55 ± 0.40 µmol, *p* < 0.0001). No difference was found in the other isoforms between HC and AD (data not shown).

### 3.3. Cytokines

**Figure 1:**
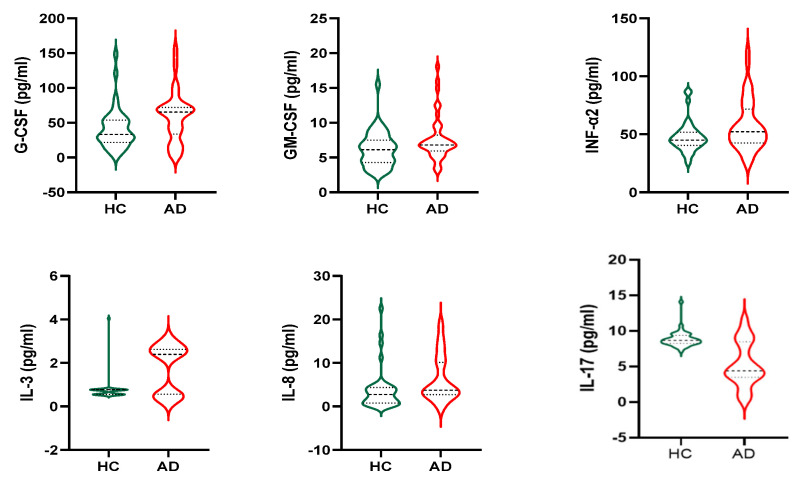
Cytokines that significantly differ between groups. HC: Healthy Control; AD: Alzheimer’s Disease.

In all populations, no difference was found in inflammatory molecules measured between genders (data not shown). Stratifying the sample population by groups of diagnosis, among all molecules examined, G-CSF, GM-CSF, INF-α2, IL-17, IL-3, and IL-8 differed significantly between HC and AD, as reported in Figure 1. AD has higher G-CSF (59.22 ± 36.71 vs. 41.32 ± 29.18 pg/mL, *p* = 0.018), GM-CSF (7.69 ± 3.25 vs. 6.19 ± 2.53 pg/mL, *p* = 0.024), INF-α2 (57.54 ± 21.93 vs. 47.57 ± 13.22 pg/mL, *p* = 0.016), IL-3 (1.75 ± 1.09 vs. 0.75 ± 0.54 pg/mL, *p* = 0.024), IL-8 (6.29 ± 5.31 vs. 3.71 ± 4.70 pg/mL, *p* = 0.024) and lower IL-17 (5.28 ± 3.12 vs. 8.86 ± 1.13 pg/mL, *p* < 0.0001) as compared with HC.

### 3.4. Exosomal miRNAs

**Figure 2:**
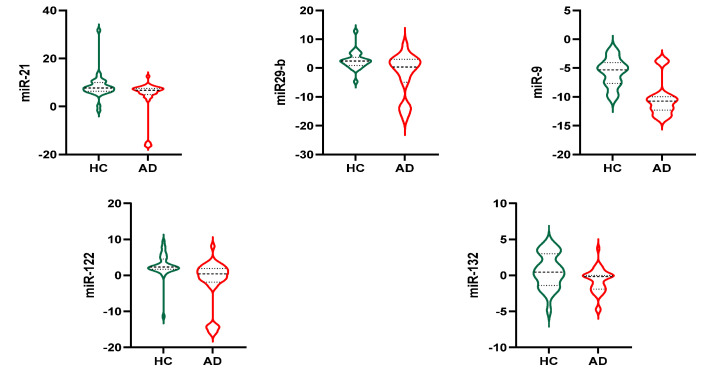
Exosomal miRNAs. HC: Healthy Control; AD: Alzheimer’s Disease.

In all populations, no difference was found in examined exosomal miRNAs between genders (data not shown). Stratifying the sample population by groups of diagnosis among all examined exosomal miRNAs (let7f5p, miR-9, miR-15a, miR-21, miR29-b, miR-122, miR-132, miR29-a, miR-128, miR-491, miR-146, miR-34, miR-874), five of them showed a statistically significant difference between groups and are reported in Figure 2. Namely, compared to HC, subjects with AD had lower miR-21 (4.1 ± 7.4 vs. 8.3 ± 5.2 *p* = 0.011), miR29-b (−2.5 ± 7.2 vs. 2.6 ± 3.0 *p* < 0.001), miR-9 (−10.4 ± 3.07 vs. −5.7 ± 2.4 *p* < 0.0001), miR-122 (−1.8 ± 6.2 vs. 2.7 ± 3.3 *p* < 0.0001), miR-132 (−0.67 ± 1.6 vs. 0.63 ± 2.3 *p* = 0.014).

**Figure 3:**
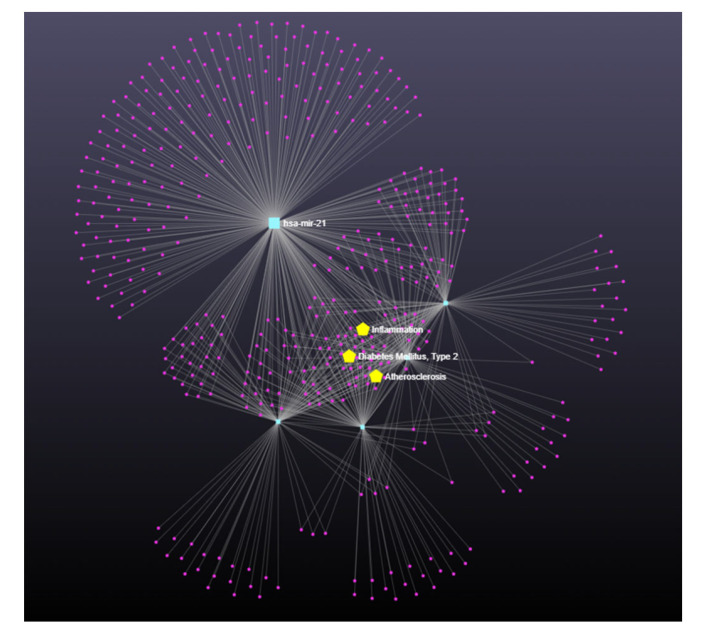
miRNet analysis (miRNet) showed that miR-9, miR-21, miR29-b, miR-122, and miR-132 are interconnected and involved in inflammation, type 2 diabetes mellitus, and atherosclerosis.

An analysis conducted with miRNet (a miRNA-centric network visual analytics platform) showed that all these miRNAs are interconnected and involved in inflammation, type 2 diabetes mellitus, and atherosclerosis (Figure 3).

Evaluating the relationship between miRNAs with inflammatory molecules in AD, we found that miR-122 significantly correlated with plasma levels of GM-CSF, INF-α2, IL-1α, IL-8, and MIP-1β (Table 2).

### 3.5. miRNA-122, Alpha-Tocopherol, and AD

In all the studied population, miR-122 correlated significantly and positively with α- tocopherol (r = 0.329, *p* = 0.006) even after correction for age and gender. A final binary logistic regression analysis showed that independent of age and gender, α-tocopherol serum levels were associated with a higher probability of having dementia (Table 3 Model 1). Such an effect was partially mediated by miR-122 (Table 3 Model 2). Such a model also showed that miR-122 was negatively and significantly associated with AD.

## 4. Discussion

The results of the study collectively show that: (1) among the vitamin E forms α tocopherol differ between groups having patients affected by AD lower levels, as compared with controls independent of age and gender; (2) among all examined inflammatory molecules some differed significantly between groups having AD higher G-CSF, GM-CSF, INF-α2, IL-3, IL-8 and lower IL-17, as compared with HC; (3) among all miRNAs examined subjects affected by AD have downregulated exosomal expression of miR-9, miR-21, miR29-b, miR-122, miR-132 as compared with HC; (4) in AD group exosomal miR-122 significantly correlates with some peripheral cytokines (GM-CSF, INF-α2, IL-1α, IL-8, and MIP-1β); (5) lower levels of serum α-tocopherol are associated with higher probability to be classified as affected by AD and miR-122 levels partially mediate such an effect.

Considering the long continuum of AD as well as its impact on human health and care services, there is an urgent need for biomarkers for preventive or curative strategies to detect AD accurately at its early stage and potentially manage it before the appearance of neurologic signs. Though each has limitations, various therapies have been developed to treat AD [22]. However, the most critical hindering factor to drug development is the lack of complete understanding of the multifactorial pathophysiology of AD.

It is well-established that inflammation and oxidative stress play a pivotal role in AD development and progression [23]. A growing body of evidence suggests the pivotal role of inflammation and related mechanisms in the pathophysiology and development of brain damage related to AD [24]. The dysregulation of cytokines is a central feature in the development of neuroinflammation and neurodegeneration in the central and peripheral nervous systems [25]. The regulation of inflammation is an active area of investigation, while oral micronutrients with antioxidant and anti-inflammatory properties supplementation is an attractive therapeutic field in AD research [11].

Among them, vitamin E is an essential micronutrient for humans, with a pivotal role in maintaining the integrity of cell membranes [26]. One of the primary functions of vitamin E is its antioxidant action, representing one of the most important lipophilic radical scavenging antioxidants, as shown in vivo studies [26]. In line with previous findings [27], here we found that α-tocopherol differed between groups with patients affected by AD lower levels compared to controls independent of gender. In detail, α-tocopherol acts as a chain-breaking antioxidant in lipoproteins and cell membranes, limiting lipid peroxidation and maintaining membrane integrity [28]. Nishida et al. [29] studied the effects of α-tocopherol deficiency in a double-mutant mouse model obtained crossing Ttpa−/−TTP knockout mice (by mutations in α-tocopherol transfer protein, α-TTP), where the lack of α-tocopherol in the brain caused oxidative stress. Then, it has been demonstrated that Aβ takes part in increasing oxidative stress, leading to lipid peroxidation and protein oxidation. In turn, also oxidative stress per se promotes Aβ production. Interestingly, the vitamin E administration in younger Tg2576 mice (from five months of age) reduced Aβ1–40 and Aβ1–42 levels and amyloid accumulation in the brain [30]. Accordingly, a higher intake of foods rich in vitamin E in humans is associated with a lower risk of dementia [31] as well as better cognitive performance [32]. In this direction, our study further supports that low serum α-tocopherol (mainly related to the introduction with diet) is associated with a higher probability of having dementia independent of multiple covariates, including age and gender. Interestingly, the ability of micronutrients to regulate the final expression of gene products via modulation of transcription and translation is now being recognized.

Modulation of miRNAs by nutrients is one pathway by which nutrition may strongly mediate gene expression [33] MiRNAs can directly regulate gene expression post-transcriptionally and indirectly influence gene expression by modulating the function of components of the epigenetic machinery (DNA methylation and histone modifications). These mechanisms interact to form a complex, bi-directional regulatory circuit regulating gene expression [34], which makes them good candidates for effective treatments of such a multifactorial disease. MiRNAs in exosomes, extracellular vesicles secreted by different cells, are essential constitutive elements that mediate intercellular communication. Recently, disease-specific miRNA profiles have been identified in AD [35]. Our study confirmed a different expression of exosomal miRNAs between AD and healthy control, which includes the downregulation of miR-21, miR29-b, miR-9, miR-122, miR-132. miR-21 mainly regulates the apoptosis and inflammatory processes in the nervous system and is also involved in astrocyte activation, glutamate toxicity, synaptic dysfunction, microglial burst activity, and remyelination [36]. Upregulated miR-21 alleviated cognitive deficits and pathological changes in APP/PS1 mice [37]. According to previous findings, the expression of miR-29 family and miR-9 [38] is significantly reduced in AD [39]. Both play an essential role in different stages of neurogenesis and neurophysiology, as well as their participation in processes such as apoptosis, inflammation, and oxidative stress [40]. miR-122 is instead a target for extensive study due to its association with cholesterol metabolism and hepatocellular carcinoma. However, no clear evidence is available for miR-122 implication in AD. miR-132, indeed, is one of the best-studied miRNAs in this field, which is among the most consistently downregulated miRNAs in AD [41]. In detail, miR-132 levels are inversely correlated with the deposition of intraneuronal hyperphosphorylated tau and extracellular amyloid aggregation in the human AD brain [42]. Altogether, our findings support the role of these identified miRNAs, downregulated in AD, not only as candidate biomarkers for AD but also because their intervention in inflammatory responses by regulating their expression may represent a novel and potential approach for treating AD. In support of such a hypothesis, the results from miRNet pathways database show that all of them are interconnected and involved in inflammation.

Most importantly, we found that miR-122 correlates positively and significantly with some peripheral cytokines (GM-CSF, INF-α2, IL-1α, IL-8, and MIP-1β). Significantly increased CSF and peripheral levels of GM-CSF (a cytokine stimulating microglial cell growth and exerting inflammatory properties) have been recently observed in subjects affected by AD and vascular dementia. It has been suggested that once secreted, GM-CSF induces programmed cell death in the brain tissue of patients with dementia [43].

Interestingly, we recently showed that an easy-to-get cytokines “signature” composed of three molecules -IFNα2, IL-1α, and TNF-α- can discriminate cognitively healthy subjects from subjects affected by AD [20] IFN-α2 has a role in the amplification of the inflammatory response inducing secretion of other cytokines and its serum level are upregulated in patients with AD. IL-1α plays a crucial role in AD pathogenesis, particularly the homozygosity for a specific IL-1α gene polymorphism at least triples the risk for the development of AD (reviewed in [44]). Again, studies in cultured human microglia display a significant role of interleukin-8 in neuroinflammation, and literature data indicate that gene polymorphism in IL-8 may affect the predisposition of AD [45]. Indeed, it has been demonstrated that micro vessels derived from AD brain express high levels of MIP-1α mRNA as well as release high levels of MIP-1α protein when compared with brain micro vessels isolated from cognitively healthy controls. Interestingly, oxidative stress alters the expression of MIP-1α in brain endothelial cells. Treatment of brain endothelial cell cultures with hydrogen peroxide or oxidatively modified low-density lipoproteins results in a dose-dependent increase in MIP-1α mRNA levels and MIP-1α release into the media. These results suggest that oxidative and lipid insults to the brain microvasculature are likely to contribute to the inflammatory milieu of the AD brain [46].

We can collectively hypothesize a pro-inflammatory role of miR-122 in AD and its relationship with oxidative stress. With this study, we show for the first time that miR-122 correlates with α-tocopherol and partially mediates the effect of such a micronutrient on the potential dementia susceptibility. miR-122, derived from a single genomic locus on chromosome 18, has a role in liver inflammation. Only a previous study showed that vitamin E deficiency resulted in reduced concentrations of miRNA-122a and miRNA-125b in rat liver [47]. miR-122-deficient mice have increased pro-inflammatory cytokines, such as IL-6 and TNF-α [48]. Still, few studies have been conducted in humans. Recent evidence shows that miR-122 can be considered a key regulator of cholesterol and fatty-acid metabolism in the adult liver, suggesting that miR-122 can be a potential therapeutic target for metabolic diseases [49]. Interestingly, impairment of energy metabolism, insulin resistance, and inflammation are three of the most critical factors implicated in the promotion of oxidative stress production that may accelerate the neurodegenerative processes leading to AD development [50]. Our data indicated a novel role tunable network in AD between α-tocopherol and miR-122 in AD, and further studies are necessary to confirm such an association. In this context, nutritional components of the diet, including vitamin E, may play a key role in the modulation of miRNA profiles and, consequently, the potential disease susceptibility. However, the limited number of subjects and the cross-sectional nature of this study represent the major limitation. Thus, more prospective cohort studies are required to assess such an association further.

## 5. Conclusions

In conclusion, our study demonstrated a potential novel property of α-tocopherol as a neuroprotectant agent in promoting AD susceptibility mediated by miR-122 through inflammation modulation. Furthermore, our findings suggest that α-tocopherol intake through the habitual diet may influence circulating inflammatory miR-122, highlighting that this aspect must be considered in the nutri-epigenomic research. Indeed, the measurement of miR-122 can also be helpful for early AD detection and may allow the development of novel targeted therapeutics.

## Figures and Tables

**Table 1:** Clinical characteristics of all sample population (n = 80) and stratified by groups. Data are expressed as mean ± standard deviation (SD). HC: Healthy Control; AD: Alzheimer’s Disease; MMSE: Mini Mental State Examination; GDS: Geriatric Depression Scale; ADL: Activity of daily living; IADL: Instrumental activity of daily living; TC: Total cholesterol; HDL-C: High Density Lipoprotein-Cholesterol; LDL-C: Low Density Lipoprotein-Cholesterol * χ^2^ = 4.114.

	Total	HC	AD	*p*
N	80	40	40	
F/M (n)	45/35	18/22	27/13	0.043 *
Age (years)	77.58 ± 3.86	76.33 ± 3.61	78.83 ± 3.74	0.003
MMSE	21.33 ± 8.32	28.83 ± 1.50	13.43 ± 4.13	<0.0001
GDS	5.03 ± 3.23	4.92 ± 2.86	5.16 ± 3.67	0.765
ADL	4.50 ± 1.56	5.53 ± 0.78	3.48 ± 1.48	<0.0001
IADL	3.83 ± 2.96	6.35 ± 1.79	1.30 ± 1.24	<0.0001
Glycemia (mg/dL)	100.5 ± 23.3	101.8 ± 24.6	99.1 ± 22.2	0.630
Creatinine (mg/dL)	1.03 ± 0.17	1.06 ± 0.23	1.0 ± 0.11	0.196
TC (mg/dL)	197.4 ± 39.9	192.8 ± 35.7	202.3 ± 43.8	0.294
HDL-C(mg/dL)	57.2 ± 15.9	57.2 ± 16.9	57.1 ± 15.0	0.986
LDL-C (mg/dL)	118.4 ± 35.2	113.3 ± 33.5	123.8 ± 36.6	0.218
Triglycerides (mg/dL)	112.1 ± 49.5	118.8 ± 48.5	125.5 ± 50.9	0.546

**Table 2:** Partial correlation between cytokines and miR-122 in AD controlled by gender (n = 40).

	Partial Correlations	
	Correlation Coefficient (r)	*p*-Value
miR-122/GM-CSF	0.389	0.019
miR-122/INF-α2	0.375	0.024
miR-122/IL-1α	0.360	0.031
miR-122/IL-8	0.375	0.024
miR-122/MIP-1β	0.354	0.034

**Table 3:** Binary logistic regression analyses to assess whether α-tocopherol levels are associated with dementia, controlling for multiple confounding factors. Gender is indicated as M = 1 and F = 0.

**Model 1**				
	**B**	**OR**	**IC 95%**	***p***
Age	0.182	1.199	1.029–1.398	0.020
Gender	0.493	1.637	0.578–4.634	0.353
α-tocopherol	−0.329	0.720	0.579–0.895	0.003
**Model 2**				
	**B**	**OR**	**IC 95%**	***p***
Age	0.175	1.191	0.989–1.434	0.065
Gender	0.047	1.048	0.326–3.363	0.938
α-tocopherol	−0.252	0.777	0.617–0.980	0.033
MiR-122	−0.215	0.806	0.654–0.995	0.045

## Data Availability

The data presented in this study are available on request from the corresponding author. The data are not publicly available due to privacy.
